# The Floating Cartilage in Joints

**Published:** 1897-08

**Authors:** W. J. Martin

**Affiliations:** Kankakee, Ill.


### THE FLOATING CARTILAGE IN JOINTS.

By W. J. Martin, V.S.,
KANKAKEE, ILL.

A few months ago I was called to an adjoining city professionally; while there I was requested by a gentleman to examine the hocks of his young stallion, two years of age. On manipulation of the left hock-joint a small, hard, nodular body could be felt on the internal aspect of the joint. The body was situated within the true joint—that is, behind the capsular ligament and synovial membrane, and was found to be floating free in the synovial fluid of the joint. The colt had never shown any symptoms of lameness, nor was the joint enlarged at any point. A diagnosis of a floating piece of cartilage within the joint, without any apparent structural changes in the articulation, was made out.

In a practice of nearly fifteen years, this is the first case of floating cartilage in a horse's joint that has come under my observation, although I have no doubt that they are more common than we are aware of, could we but detect their presence in joints. There is no doubt in my mind, that in the horse loose pieces of cartilage in joints are often the cause of many cases of obscure lameness, such as "cramp" of the hind limbs of young horses, in which cases the limbs are often most rigidly locked, admitting of neither tension, flexion, nor rotation. Loose pieces of cartilage may also be the cause of acute arthritis when, owing to the amount of swelling present in the joint, detection of the pieces

**It will more firmly cement the fraternal feelings that bind us: North, South, East, West.**

may be overlooked, or they may become fastened to some part of the joint by the resulting inflammation.

Floating cartilage in joints is quite common in man, and is often the cause of so serious a disorganization of joints as to render amputation necessary. The composition of these hard bodies found in the joints of many vary, some being composed of numerous layers of compressed fibrin, while others are made up of portions of ossifying cartilage, and in reality are true bone. The causes which give rise to the presence of these loose pieces of cartilage in joints are various, perhaps the chief one is that as the articular cartilage derives most of its growth and nourishment by imbibition from the network of fine bloodvessels that terminate near the shaft of long bones which go to make up an articulation, any redundance of their cartilage might be accounted for by a too rapid growth of the cartilage within the joint at a certain point, which becoming loosened from the parent bone, floats off in the synovial fluid in the joint. Again, the formation of these morbid growths in joints is due to other causes, any one of which acting separately may give rise to them.

**You will honor our hard-working efficient Secretary.**

In some cases clots of fibrin may become solidified together; the presence of this clotted fibrin may be due to inflammatory exudation taking place within the joint. In other cases, blood may become extravasated into a synovial hinge, and there, becoming organized, break off and fall into the joint-cavity; or it may remain attached by a pedicle, and thus hang pendulous in the joint until it becomes caught between the ends of the articulating bones forming the joint, thus giving rise to sudden and severe lameness. In other cases the cells of the villous portion of the synovial membrane, which contain a few cartilage cells, may become enlarged from acute or subacute arthritis, and drop into the cavity of the joint. Sometimes a piece of the articular cartilage, together with a small piece of the bone, may, after an injury, be forced into the joint, causing inflammation and severe lameness. In osteo-arthritis the edges of the articular cartilage become much thickened and calcified, and sometimes these break off and fall into the joint. The size of these bodies found in human joints may vary from the size of a millet-seed to that of a walnut, and as many as sixty of them have been removed from a single joint.

In the human patient the symptoms of loose pieces of cartilage when they become caught between the ends of the long bones of a joint are most characteristic, such as sudden and acute pain in the joint, often extension, flexion, or rotation is impossible and the patient will often fall to the ground if unsupported. The offending body may sometimes be seen producing a bulging outward of the skin covering the joint when situated superficial, or if deep-seated within the joint it may be felt as a hard nodular body, which will sometimes slip from one side of the joint to the other. There appears to me no good reason why a disease which causes so much pain and suffering in the human species might not also be the cause of many pathological evils in the equine species. I think that this is a subject that may well engage the attention of veterinary pathologists, to render clear to us some of the lesions due to floating cartilage in equine joints.

